# The knowledge, attitudes and perceptions of physiotherapists and chiropractors in South Africa

**DOI:** 10.4102/sajp.v80i1.1922

**Published:** 2024-02-29

**Authors:** Micaela Ravidutt, Sonill Mahara

**Affiliations:** 1 Department of Chiropractic, Durban University of Technology, Durban, South Africa; 2 Department of Physiotherapy, University of KwaZulu-Natal, Durban, South Africa

**Keywords:** chiropractors, physiotherapists, physiotherapy, chiropractic, knowledge, attitudes, perceptions, collaboration

## Abstract

**Background:**

Effective healthcare delivery occurs when health professionals collaborate and provide holistic, patient-centred care. Physiotherapists and chiropractors treat a common range of patients with an overlap in their scope of practice and modalities because of typical healthcare roles that could lead to ‘perceived’ animosity.

**Objectives:**

To assess the knowledge, attitudes, and perceptions of qualified chiropractors and physiotherapists regarding each other’s practice.

**Method:**

A cross-sectional survey using an online questionnaire and analysed descriptively.

**Results:**

Participants were chiropractors (*n* = 116) and physiotherapists (*n* = 190). Chiropractors achieved a mean knowledge score of 75.7%, with physiotherapists at 59.7% on the assessments of each other’s patients; an average score of 85.3% and 72.0% respectively, on knowledge of treatment modalities; knowledge score of 82.4% and 77.3% respectively, on the conditions treated by the other professional. A total of 82.8% (*n* = 96) of chiropractors and 70.0% (*n* = 133) of physiotherapists indicated the other professionals’ competence in treating neuromusculoskeletal conditions. Inter-professional referrals occurred between 81.9% of chiropractors (*n* = 95) and 55.3% of physiotherapists (*n* = 105). Chiropractors (69.0%, *n* = 80) and physiotherapists (55.3%, *n* = 105) wanted to collaborate to manage patients.

**Conclusion:**

In the surveyed population in South Africa, chiropractors and physiotherapists had good knowledge, positive attitudes and perceptions of each other’s practices, especially in the private sector.

**Clinical implications:**

Inter-professional collaboration between chiropractors and physiotherapists should be encouraged so that healthcare delivery can be holistic and patient-centred for better clinical outcomes.

## Introduction

In current financial circumstances and especially during the coronavirus disease 2019 (COVID-19) pandemic, the workloads of healthcare professionals have drastically increased, requiring inter-professional interactions and collaboration for patients to receive effective and efficient healthcare services. The scope of physiotherapists and chiropractors includes non-invasive therapy to treat pain and improve joint movement and function (Carlesso et al. 2014; Slabbert 2014; Thondhlana 2018). Although there are specified and regulated scopes of practice for physiotherapists and chiropractors in South Africa, the skills and modalities of each professional sometimes overlap. This could lead to competition for patients, leading to ‘perceived animosity’ between these two professionals, especially in private healthcare in South Africa. Currently, there are no studies done internationally on ‘perceived animosity’ between chiropractors and physiotherapists.

### Physiotherapy in South Africa

In South Africa, physiotherapy is studied at eight institutions, namely the University of Cape Town, University of the Free State, University of KwaZulu-Natal, University of Pretoria, Stellenbosch University, University of Western Cape, University of the Witwatersrand, and Sefako Makgatho Health Science University (South African Society of Physiotherapy 2023a). The course is 4 years; when completed, there is an additional year of ‘compulsory community’ service. The Health Professions Council of South Africa (HPCSA) regulates physiotherapy practice by *Act 56 of 1974*, which states the scope of physiotherapists ‘as a supplementary service to medicine’, which includes orthopaedics, paediatrics, geriatrics, neurology, neurosurgery, respiratory disease, cardiovascular diseases, obstetrics, gynaecology, intensive care services, sports medicine and rehabilitation’ (HPCSA 2022). Physiotherapists use modalities like ‘mobilisation and manipulation of joints, massage, acupressure, dry needling, exercise programmes, relaxation techniques, hydrotherapy, biofeedback, electrotherapy equipment, thermal therapy, cryotherapy, traction, assistive devices and patient education’ (Carlesso et al. 2014; South African Society of Physiotherapy 2023b). In addition, the Chartered Society of Physiotherapy (2022) defines physiotherapy as a healthcare profession that facilitates the restoration of movement and function when a person is affected by injury, illness or disability through movement, exercises, manual therapy, education, and advice. This is achieved by using electro- and physical therapy to rehabilitate functional disabilities and pain with functional or transcutaneous electrical stimulation, interferential therapy, heat, ice, ultrasound, massage, manipulative therapy, acupuncture, therapeutic exercises, orthotic, prosthetic devices and splinting, which have shown positive benefits (Chartered Society of Physiotherapy 2022).

### Chiropractic in South Africa

Chiropractic is defined as a health profession specialising in the diagnosis, treatment and prevention of mechanical disorders of the musculoskeletal system and the effects of these disorders on the function of the nervous system and general health (Chiropractic Association of South Africa 2022a; Jones-Harris 2010; World Federation of Chiropractic 2001). In South Africa, chiropractic is studied at the Durban University of Technology (DUT) or the University of Johannesburg (Chiropractic Association of South Africa 2022b). The course lasts 6 years, with chiropractors graduating with a Master’s Degree in Health Sciences. (Chiropractic Association of South Africa 2022b; Durban University of Technology 2023). On completing the degree, chiropractors must complete 675 h of internship. This comprises 75 academic and 600 work experience hours, after which they register with the Allied Health Professions Council of South Africa (AHPCSA). The AHPCSA regulates the scope of chiropractic in South Africa through the *Associated Health Service Professions Act of 1982*. This act allows chiropractors to perform ‘a physical examination with or without reading and interpreting X-ray plates to diagnose physical defects, illness or deficiency, and treat or prevent physical defects or disease of the spinal, pelvic, spino-visceral and general neuromusculoskeletal disorders’ (AHPCSA 2018). Chiropractors use joint manipulation, electrotherapy, hydrotherapy, thermal therapy, cryotherapy, massage, traction, vibration therapy, exercises, immobilisation therapy, acupressure, neuro-muscular reflex therapy, nutritional and supplementation advice when managing patients (AHPCSA 2018; Carlesso et al. 2014; Hartvigsen & French 2020).

### Overview

From the above definitions and scopes for physiotherapists and chiropractors, it seems both professionals have similar modalities and patient outcomes and are comparable (Hunter 2004; Ryan, San Too & Bismark 2018). Although both professionals practise in differing clinical paradigms, the overlap of skills and modalities can make these professions complementary (Carlesso et al. 2014; Hunter 2004; Naidoo & Bühler 2009).

Differences in professional awareness between the varying skills of physiotherapists and chiropractors in various countries have been reported (Paul & Mullerpatan 2015). A study by Westin et al. (2013) investigated the general practitioners’ opinions and perceptions of chiropractic in Norway and Sweden, and found that general practitioners believed that chiropractors are proficient in treating musculoskeletal ailments. A study to assess the attitudes of North American orthopaedic surgeons towards chiropractors by Busse et al. (2011) found that attitudes towards chiropractic varied between ‘very positive and extremely negative’. Factors that influenced negative attitudes included the older age of the surgeons, the acceptance of evidence-based literature or the media as a reference for information about the chiropractic profession. Positive attitudes of chiropractors were related to these surgeons interacting with chiropractors. Another study to determine the knowledge and perceptions of Saudi Arabian physicians found that 51.0% had some knowledge of physiotherapy, but 58.0% had negative perceptions about the profession (Al-Eisa et al. 2016). Thondhlana (2018) found that general practitioners in Harare, Zimbabwe, had a fair knowledge of chiropractic services because of their inter-professional experience, educational training, and collaboration with chiropractors. Studies conducted in Mumbai and Punjab, India, found that general practitioners adequately understood physiotherapy and its role in healthcare (Agni & Battin 2017; Vardhan et al. 2018).

Differences in the knowledge of these two professions have also been reported in the general public. In India, 90% of healthcare workers and 78% of the general public knew about physiotherapy services but did not know that physiotherapists also worked in the cardiovascular, pulmonary, oncology, obstetrics, and gynaecology wards (Doshi et al. 2017). An international study by Paul and Mullerpatan (2015) on physiotherapy awareness revealed that 35.0% of their sample resided in urban and semi-urban regions in the United Kingdom, and 29.5% of Australians had knowledge of physiotherapy. However, 85.0% residing in a Nigerian village did not know physiotherapy.

The perception towards each other’s profession has also been studied. Naidoo and Bühler (2009) investigated the knowledge, attitudes and perceptions of chiropractic and physiotherapy students towards each other in KwaZulu-Natal, South Africa. Their study revealed that chiropractic and physiotherapy students had comparable levels of knowledge, but chiropractic students had ‘more positive perceptions and attitudes toward physiotherapy’ compared to physiotherapists to chiropractic. A study by Hunter (2004) of South African physiotherapists’ attitudes and perceptions of the chiropractic profession showed that 66% of physiotherapists lacked knowledge of chiropractors, and 88% indicated that improved communication and cooperation between chiropractors and physiotherapists would benefit professionals and patients.

In South Africa, physiotherapy is integrated into both the public and private health sectors (South African Society of Physiotherapy 2023b). However, current legislation restricts chiropractors to the private health sector (Davies 2018; Naidoo & Bühler 2009). There is, therefore, a challenge in private practice because many health professionals, like physiotherapists and chiropractors, ‘compete’ for patients, leading to ‘perceived animosity’ between them (Ellapen et al. 2018). Inter-professional collaboration with knowledge, positive attitudes and perceptions between physiotherapists and chiropractors must be improved and enhanced. However, limited and sometimes conflicting studies exist between physiotherapists and chiropractors (Hunter 2004; Langworthy & Smink 2000; Naidoo & Bühler 2009; Slabbert 2014). This makes it necessary and prudent for this preliminary study of these professionals’ knowledge, attitudes and perceptions towards each other. Therefore, the primary aim of our study was to determine the knowledge, attitudes and perceptions of qualified chiropractors and physiotherapists in South Africa regarding each other’s professional practice. The secondary aim was to analyse the data to encourage and enhance an inter-professional, amicable, positive acceptance and collaboration between South African physiotherapists and chiropractors.

## Research methods and design

### Design of the study

This cross-sectional point prevalence study was conducted with physiotherapists and chiropractors in South Africa using an online self-designed questionnaire. Slabbert (2014) granted permission to adapt his questionnaire for the draft questionnaire to align with the aims and objectives of our study. The draft questionnaire included questions from other questionnaires (Hunter 2004; Palmer 2008). The questionnaire consisted of 22 closed-ended and 2 open-ended questions that extrapolated information of physiotherapists’ and chiropractors’ knowledge, attitudes and perceptions regarding each other’s professional practice and inter-professional relationships. The draft questionnaire was the pilot study and was emailed to five physiotherapists and five chiropractors who did not participate in the main study. They reviewed the questionnaire and indicated whether the questions were appropriate to meet the study’s aims and objectives. The final questionnaire was designed based on their feedback and suggestions (Online Appendix 1 and Online Appendix 2). The questionnaire consisted of 7 knowledge questions, 11 Likert scale questions on attitudes, 11 Likert scale questions on perceptions and 10 questions on inter-professional relationships. The AHPCSA distributed the questionnaires to the chiropractors via email, and to promote participation, the Chiropractic Association of South Africa also distributed the questionnaires electronically. The physiotherapy questionnaire was initially distributed to the physiotherapists via email using Medpages. However, because of a poor response rate from the physiotherapists, the authors requested the assistance of the South African Society of Physiotherapy to distribute the online questionnaires to the physiotherapists. The questionnaires did not identify the participant’s professional association. The only inclusion criterion was that practitioners must practise and be registered with their relevant councils – HPCSA for physiotherapists and AHPCSA for chiropractors. Only those who provided online informed consent could participate and access the questionnaire. An online link was established for each profession (physiotherapists: https://dut.questionpro.com/t/AQrgIZlUg6 and chiropractors: https://dut.questionpro.com/t/AQrgIZlUdT).

### Sample size calculation

Based on the statistician’s recommendation, the sample size for each profession was calculated separately using Cochran’s sample size for categorical data:

$$=\frac{{\left(t\right)}^{2}(p)(q)}{{\left(d\right)}^{2}}$$ [Eqn 1]

Where,

*t* = value for a selected alpha value of 0.25 in each tail = 1.96

(*p*)(*q*) = estimate of variance = 0.25

*d* = acceptable margin of error = 0.085

Therefore:

$$n0=\frac{{\left(1.96\right)}^{2}(0.5)(0.5)}{{\left(0.085\right)}^{2}}=133$$ [Eqn 2]

$$n1=\frac{n0}{1+\frac{n0}{Population}}$$ [Eqn 3]

Therefore, in a population of 2987 physiotherapists, who were registered with Medpages, the minimum sample required was as follows:

$$n1=\frac{133}{1+\frac{133}{2987}}=128\;physiotherapists$$ [Eqn 4]

Therefore, in a population of 896 chiropractors, who were registered with the AHPCSA, the minimum sample required was as follows:

$$n1=\frac{133}{1+\frac{133}{896}}=116\;chiropractors$$ [Eqn 5]

The minimum sample size required for our study comprised 116 chiropractors and 128 physiotherapists. There were 116 chiropractors and 190 physiotherapists who had participated in our study. The chiropractic response rate was 12.95% (116/896) of the total sample population of chiropractors and 100% (116/116) of the required sample population. The physiotherapy response rate was 6.36% (190/2987) of the total sample population of physiotherapists and 100% of the required sample population. All 190 responses were included in our study.

### Data analysis

A statistician extracted the data from the questionnaires and analysed it using Statistical Package for the Social Sciences SPSS^®^ Version 25.0. Descriptive statistics were used with the open-ended questions grouped into themes by author, M.R., based on the responses and closed-questions depicted in tables using percentages.

### Ethical considerations

The DUT Chiropractic Departmental Research Committee and the Faculty Research Committee approved the research proposal. Ethical clearance was obtained from the Institutional Research Ethics Committee (IREC) of DUT on 05 March 2021 (Ethical Clearance Number: IREC 123/20).

## Results

The participants of our study were physiotherapists (*n* = 190) and chiropractors (*n* = 116). The questionnaires were received anonymously.

### Demographics

Participant demographics are tabulated in Table 1.

**TABLE 1:** Demographics of participants.

Demographics	Chiropractors		Physiotherapists	
	%	*n*	%	*n*
**Age (in years)**				
< 26	6.0	7	3.7	7
26–35	49.1	57	33.2	63
36–45	25.9	30	32.1	61
46–55	17.2	20	21.1	40
56–65	0.9	1	7.4	14
> 65	0.9	1	2.6	5
**Gender**				
Male	41.4	48	13.7	26
Female	58.6	68	86.3	164
**No. of years qualified**				
< 6	42.2	49	9.5	18
6– < 11	20.7	24	21.1	40
11– < 16	12.1	14	12.6	24
16– < 21	14.7	17	17.4	33
21–30	8.6	10	24.7	47
> 30	1.7	2	14.7	28
**Practice sector**				
Public	1.7	2	6.8	13
Private	94.0	109	87.4	166
Both public and private	4.3	5	5.8	11
**Practice location**				
Suburb	87.1	101	73.7	140
Central business district	10.3	12	17.9	34
Rural	2.6	3	8.4	16
**Practice type**				
Single practice	56.0	65	35.8	68
Duo-practice	32.8	38	21.6	41
Group-practice	11.2	13	42.6	81
**Province in which practice is located**				
Eastern Cape	1.7	2	3.7	7
Free State	1.7	2	3.2	6
Gauteng	50.0	58	35.3	67
KwaZulu-Natal	25.9	30	13.2	25
Limpopo	1.7	2	5.3	10
Mpumalanga	2.6	3	4.2	8
Northern Cape	0.0	0	1.1	2
North West	0.0	0	2.6	5
Western Cape	16.4	19	31.6	60

*Source:* Ravidutt, M., 2022, ‘The knowledge, attitudes and perceptions of qualified chiropractors and physiotherapists in South Africa regarding the other professional practice’, Master’s thesis, Dept. of Chiropractic, Durban University of Technology, viewed 13 November 2023, from https://openscholar.dut.ac.za/handle/10321/4993

The physiotherapy participants were 86.3% (*n* = 164) female and 13.7% (*n* = 26) male, with 33.2% (*n* = 63) between the ages of 26 and 35 years. Qualification ranged from 21 to 30 years for 24.7% of physiotherapists (*n* = 47), with 42.6% (*n* = 81) practising within a group practice having more than two physiotherapists and 87.4% (*n* = 166) practising in private health care. About 73.7% of physiotherapists (*n* = 140) practised within a suburb. The percentage of chiropractors that were female was 58.6% (*n* = 68), and 41.4% (*n* = 48) were male, with 49.1% (*n* = 57) between the ages of 26 and 35 years. Qualification of chiropractors for less than 6 years was 42.2% (*n* = 49), and 56.0% (*n* = 65) practised individually, with 94.0% (*n* = 109) working in private health care. About 87.1% of chiropractors (*n* = 101) practised within a suburb.

### Knowledge of the other profession

Both professionals had poor knowledge of registered practitioners in South Africa. Only 11.2% of chiropractors (*n* = 13) and 18.4% of physiotherapists (*n* = 35) were aware of the number of registered physiotherapists and chiropractors in the country, respectively. A large percentage of chiropractors (93.1%, *n* = 108) knew that physiotherapists graduate with a Bachelor’s Degree, whereas 26.3% of physiotherapists (*n* = 50) knew that chiropractors graduate with a Master’s Degree. Chiropractors achieved a knowledge score of 73.5% and physiotherapists 77.8%, indicating good knowledge about the institutions that offered physiotherapy and chiropractic programmes in South Africa. Many chiropractors (94.0%, *n* = 109), compared to a few physiotherapists (37.4%, *n* = 71), knew the duration of the physiotherapy and chiropractic courses, respectively. The chiropractors and physiotherapists scored 75.7% and 59.7%, respectively, regarding their knowledge of the assessments that the other professionals could conduct on patients. Both professionals demonstrated a good understanding of the treatment modalities offered by the respective professional, with chiropractors having a knowledge score of 85.3% and physiotherapists 72.0%. Similarly, both professionals demonstrated good knowledge regarding the conditions the other professional could treat, scoring 82.4% and 77.3% by chiropractors and physiotherapists, respectively.

### Attitude towards the other profession

The results are tabulated in Table 2.

**TABLE 2:** Comparing the attitudes of chiropractors and physiotherapists regarding each other in South Africa.

Chiropractors	Physiotherapists
Agreed on: Physiotherapists are competent in musculoskeletal and neurological examination (75.0%, *n* = 87). Physiotherapists are competent in managing and treating patients with neuromusculoskeletal conditions (82.8%, *n* = 96). Chiropractic and physiotherapy are complementary professions (85.3%, *n* = 99). Physiotherapy is effective for some patients (94.0%, *n* = 109). Some chiropractors would like to work closely with the physiotherapy profession (69.0%, *n* = 80). Some chiropractors already work closely with a physiotherapist (54.3%, *n* = 63).	Agreed on: Chiropractors are competent in musculoskeletal and neurological examination (72.1%, *n* = 137). Chiropractors are competent in managing and treating patients with neuromusculoskeletal conditions (70.0%, *n* = 133). Chiropractic and physiotherapy are complementary professions (72.1%, *n* = 137). Chiropractic is effective for some patients (91.1%, *n* = 173). Some physiotherapists would like to work closely with the chiropractic profession (55.3%, *n* = 105).
Disagreed on: Physiotherapists can perform the same treatment as chiropractors without additional training (93.1%, *n* = 108). Physiotherapy treatments have been shown to be superior to chiropractic (84.5%, *n* = 98). Physiotherapy causes more harm than good (90.5%, *n* = 105).	Disagreed on: Chiropractors can perform the same treatment as physiotherapists without additional training (46.8%, *n* = 89). Physiotherapy treatments have been shown to be superior to chiropractic (62.6%, *n* = 119). Chiropractic and physiotherapy are similar professions (47.4%, *n* = 90). Chiropractic causes more harm than good (62.6%, *n* = 119). Some physiotherapists already work closely with a chiropractor (54.2%, *n* = 103).

*Source:* Ravidutt, M., 2022, ‘The knowledge, attitudes and perceptions of qualified chiropractors and physiotherapists in South Africa regarding the other professional practice’, Master’s thesis, Dept. of Chiropractic, Durban University of Technology, viewed 13 November 2023, from https://openscholar.dut.ac.za/handle/10321/4993

Seventy-five per cent of chiropractors (*n* = 87) and 72.1% (*n* = 137) of physiotherapists indicated that the other profession is competent in musculoskeletal and neurological examination. Similarly, 82.8% (*n* = 96) of chiropractors and 70.0% (*n* = 133) of physiotherapists agreed that the other profession is competent in treating neuro-musculoskeletal conditions. Chiropractors (85.3%, *n* = 99) and physiotherapists (72.1%, *n* = 137) supported both professions being complementary, and 94.0% (*n* = 109) of chiropractors compared to 91.1% (*n* = 173) of physiotherapists indicated that the other profession’s treatments were effective. Also, 93.1% (*n* = 108) believed that physiotherapists needed additional training to perform equivalent treatments as chiropractors, and 90.5% (*n* = 105) indicated that physiotherapists did not cause harm to the patients. On the contrary, 46.8% (*n* = 89) of physiotherapists indicated that chiropractors needed additional training to perform physiotherapy treatments, and 62.6% (*n* = 119) of physiotherapists did not believe that chiropractors cause more harm than good. Additionally, 47.4% (*n* = 90) of physiotherapists did not believe that chiropractic and physiotherapy are similar professions, and 62.6% (*n* = 119) did not view physiotherapy as superior to chiropractic. There was support from physiotherapists (55.3%, *n* = 105) and chiropractors (69.0%, *n* = 80) to collaborate.

### Perceptions about each other

The perceptions are tabulated in Table 3.

**TABLE 3:** Perceptions of chiropractors and physiotherapists in South Africa regarding each other.

Chiropractors	Physiotherapists
Agreed on: Physiotherapy is a secondary healthcare service (68.1%, *n* = 79). Physiotherapy is accessible to all members of the public (81.9%, *n* = 95). Physiotherapy is included in Medical Aid cover (98.3%, *n* = 114). Physiotherapy is needed in South Africa (94.0%, *n* = 109). Physiotherapy plays an important role in the healthcare system (97.4%, *n* = 113). Physiotherapy is based on scientific evidence (92.2%, *n* = 107). Physiotherapy is registered with the Health Professions Council of South Africa (95.7%, *n* = 111).	Agreed on: Chiropractic is an alternative healthcare service (72.6%, *n* = 138). Chiropractic is included in Medical Aid cover (58.9%, *n* = 112). Chiropractic is needed in South Africa (60.5%, *n* = 115). Chiropractic plays an important role in the healthcare system (57.9%, *n* = 110). Chiropractic is based on scientific evidence (51.6%, *n* = 98). Chiropractic is registered with the Allied Health Professions Council of South Africa (77.4%, *n* = 147).
Disagreed on: Physiotherapy is an alternative healthcare service (62.1%, *n* = 72). Physiotherapy is registered with the Allied Health Professions Council of South Africa (94.8%, *n* = 110).	Disagreed on: Chiropractic is affordable (38.4%, *n* = 73). Chiropractic is registered with the Health Professions Council of South Africa (58.4%, *n* = 111).

*Source:* Ravidutt, M., 2022, ‘The knowledge, attitudes and perceptions of qualified chiropractors and physiotherapists in South Africa regarding the other professional practice’, Master’s thesis, Dept. of Chiropractic, Durban University of Technology, viewed 13 November 2023, from https://openscholar.dut.ac.za/handle/10321/4993

The perceptions of chiropractors towards physiotherapy were that it is a secondary healthcare service (68.1%, *n* = 79), is accessible to all members of the public (81.9%, *n* = 95), is included in medical aid cover (98.3%, *n* = 114), is needed in South Africa (94.0%, *n* = 109), plays a vital role in the healthcare system (97.4%, *n* = 113), is based on scientific evidence (92.2%, *n* = 107).

The perceptions of physiotherapists regarding chiropractic were that it is an alternative healthcare service (72.6%, *n* = 138), included in medical aid cover (58.9%, *n* = 112), needed in South Africa (60.5%, *n* = 115), important for the healthcare system (57.9%, *n* = 110), based on scientific evidence (51.6%, *n* = 98). It was also noted that 38.4% of physiotherapists (*n* = 73) did not believe chiropractic was affordable.

### The influence of knowledge, attitudes and perceptions on inter-professional relationships

Good knowledge, positive attitudes and perceptions between professionals determine inter-professional relationships. When these attributes are positive between professionals, there are good inter-professional relationships. Inter-professional relationships between physiotherapists and chiropractors are tabulated in Table 4. The responses from this study indicated that 81.9% of chiropractors (*n* = 95) referred their patients to physiotherapists compared to 55.3% of physiotherapists (*n* = 105) referring their patients to chiropractors. Referrals occurred when the physiotherapist or chiropractor had limited knowledge of the patient’s symptom(s) or condition or when these cross-referrals would benefit the patient. A total of 89.7% of chiropractors (*n* = 104) and 81.6% of physiotherapists (*n* = 155) indicated that inter-professional relationships between both professions would benefit the patient. Similarly, 82.8% of chiropractors (*n* = 96) and 70.5% of physiotherapists (*n* = 134) agreed that physiotherapy would benefit from such inter-professional relationships. It was also noted that 83.6% of chiropractors (*n* = 97) and 74.7% of physiotherapists (*n* = 142) thought these inter-professional relationships were beneficial to chiropractic.

**TABLE 4:** Inter-professional relationships between chiropractors and physiotherapists in South Africa.

Inter-professional relationships	Chiropractors		Physiotherapists	
	*n*	%	*n*	%
**Referral of patients to the other professional**	95	81.9	105	55.3
**Reasons for referral to the other professional**				
Condition of the patient	-	-	-	-
Best interests of the patient	-	-	-	-
Limited scope of practice	-	-	-	-
Collaborative treatment	-	-	-	-
**Received patient referrals from the other professional**	81	69.8	61	32.1
**Benefit from inter-professional collaboration**				
For patient	104	89.7	155	81.6
For physiotherapy profession	96	82.8	134	70.5
For chiropractic profession	97	83.6	142	74.7

*Source:* Ravidutt, M., 2022, ‘The knowledge, attitudes and perceptions of qualified chiropractors and physiotherapists in South Africa regarding the other professional practice’, Master’s thesis, Dept. of Chiropractic, Durban University of Technology, viewed 13 November 2023, from https://openscholar.dut.ac.za/handle/10321/4993

## Discussion

The study was designed to determine the knowledge, attitudes and perceptions between physiotherapists and chiropractors to each other. There is a strong association between knowledge, attitudes and perceptions, as perceptions are influenced by one’s knowledge, which determines one’s attitudes, and poor knowledge may result in negative attitudes and incorrect perceptions (Zhu & Xie 2015). Based on the results of our study, it can be seen that chiropractors demonstrated good knowledge, positive attitudes and perceptions towards physiotherapists. Most chiropractors knew the qualification that physiotherapists obtain in South Africa, but a limited number of physiotherapists knew that chiropractors received a Master’s degree or ‘doctor’ title upon graduation. This correlated with physiotherapists having poor knowledge, whereas chiropractors having a good understanding of the number of years required to study for their relevant degree. This is supported by other studies showing physiotherapists had poor knowledge of the qualification obtained by chiropractors as well as other health professionals also having poor knowledge regarding a chiropractor’s qualification in South Africa (Heslop 2008; Hunter 2004; Maharaj 2009; Naidoo 2008). This may suggest that these health professionals may view chiropractors as less qualified than they are, which may be attributed to limited inter-professional awareness and interaction of these professionals with chiropractic because chiropractic is a small profession in South Africa (Heslop 2008). Both professionals were well aware about the institutions that offered physiotherapy and chiropractic in South Africa, as no single institution offered both qualifications. The authors postulate that this could be attributed to learners researching career choices during high school. The physiotherapy results are considerably higher than those obtained by South African paediatricians and provincial and national health portfolio committee members in South Africa (Heslop 2008; Maharaj 2009).

Both professionals had sufficient knowledge of the conditions treated and modalities offered by the other professional. This is a positive outcome as it reflects that the professionals recognise each other’s role in treating patients and providing healthcare. The physiotherapy results in our study are higher than those obtained by Hunter (2004), which highlights that over the years, there has been an improvement in the physiotherapists’ knowledge that chiropractors can treat chronic pain disorders, temporomandibular joint disorders, sports injuries, joint and ligament sprains, muscle strains, and disc herniation. Furthermore, there has been improved awareness since the 2004 study that chiropractors can perform massage, cryotherapy, thermotherapy, acupuncture and joint mobilisation, and provide nutritional and ergonomic advice. In contrast, chiropractors in this study demonstrated better knowledge of the physiotherapists’ professional practice than those of physiotherapists of chiropractors. This is contrary to the study by Naidoo and Bühler (2009), in which physiotherapy and chiropractic students demonstrated equal knowledge of the other profession. The authors of this study postulate that this discrepancy may be because of students having better knowledge since they choose their careers at this stage, and professional competition is not a factor. However, once qualified and in clinical practice, health professionals compete with each other, and chiropractors being relatively small in number compared to physiotherapists makes chiropractors more aware of their competitors.

In this study, both professionals agreed that the other was competent in examining, managing and treating musculoskeletal and neurological conditions. They were complementary professionals, and their treatment modalities were effective for patients. This augurs well for inter-professional collaboration. Studies of different health professionals by Heslop (2008), Hunter (2004), Maharaj (2009), Naidoo (2008), and Palmer (2008) reported similar results regarding the competency of chiropractors in the examination and treatment of neuro-musculoskeletal complaints. Slabbert (2014) also said that most chiropractors and physiotherapists believed that both professions are complementary. The physiotherapy findings in our study are higher than those reported by Hunter (2004), Langworthy and Smink (2000) and Maharaj (2009), in which various health professionals agreed that chiropractic and physiotherapy are complementary professions. In terms of the effectiveness of the chiropractic treatments, our physiotherapy result is higher than those obtained by various health professionals in studies conducted by Busse et al. (2011), Heslop (2008), Maharaj (2009), Naidoo (2008), and Palmer (2008). The participants in our study also supported that physiotherapy or chiropractic modalities and treatments did not cause harm and were beneficial for patients. The physiotherapy results in our study are remarkably higher than the findings of Heslop (2008) and Maharaj (2009). Our results support that both professions highly regard the skills and expertise of the other profession. The perception of physiotherapists towards chiropractors was that chiropractic is an alternative healthcare service, but chiropractors did not support physiotherapy being an alternative healthcare service. This concurs with Hunter (2004) and Maharaj (2009), in which the majority of physiotherapists and provincial and national health portfolio committee members, respectively, indicated that chiropractic is an alternative healthcare service. However, less than half of the pharmacists in a South African study by Palmer (2008) and physiotherapists, osteopaths and manual therapists in a Netherlands study by Langworthy and Smink (2000) agreed that chiropractic is an alternative healthcare service. Perhaps this discrepancy is because of chiropractic being classified as complementary and alternative medicine, whereas physiotherapy is considered complementary because it is used as a supplementary service to medicine (Health Professions Council of South Africa 2022; McCubbin 2017). A limited number of chiropractors in this study perceived physiotherapy as a secondary healthcare service because, in some countries, patients require a referral from a general practitioner to seek physiotherapy care (Agni & Battin 2017; Vardhan et al. 2018). In South Africa, however, physiotherapy is a primary healthcare service and physiotherapists can treat patients without a referral from a doctor (South African Society of Physiotherapy 2023c). Perhaps this may be why chiropractors refer to physiotherapy as a ‘secondary healthcare service’. Most chiropractors believed that physiotherapy is accessible to all members of the public and is included in medical aid cover. Similarly, most physiotherapists knew that chiropractic services are included in medical aid coverage but perceived the medical aid rates for chiropractic care to be expensive. Fewer than 50% of the physiotherapists, osteopaths and manual therapists in a Netherlands study believed that chiropractic is accessible to all and is expensive (Langworthy & Smink 2000). The results of the study indicate that both professions know that medical aid schemes cover chiropractic and physiotherapy; however, less than half of the physiotherapists did not believe that chiropractic is affordable, which could potentially hinder inter-professional relationships and patient referrals.

Both professionals perceived the other to be based on scientific evidence, which suggests mutual respect and acceptance of the other profession. When compared to studies by Hunter (2004), Langworthy and Smink (2000) and Palmer (2008), only a small percentage of various healthcare practitioners believed that chiropractic lacked scientific evidence. Maharaj (2009) found that most South African provincial and national health portfolio committee members did not believe chiropractic lacked scientific background. A study by Al-Eisa et al. (2016) found that a low number of Saudi Arabian physicians felt that physiotherapy lacked a scientific basis. Both physiotherapists and chiropractors acknowledged that inter-professional relationships between chiropractors and physiotherapists would benefit patients, suggesting that both professionals value each other’s role in healthcare. Similar results were noted by Hunter (2004), where the majority of physiotherapists believed that ‘good cooperation’ between these professionals would benefit both professions and their patients. Naidoo and Bühler (2009) also noted that most final-year chiropractic students agreed that patients would benefit more from inter-professional collaboration between chiropractors and physiotherapists. Both professions indicated their interest in collaborating with the other profession. The results compare favourably with Naidoo and Bühler (2009), in which chiropractic and physiotherapy students reported being inclined to collaborate with the other profession. Our findings support mutual respect between physiotherapists and chiropractors in healthcare provision and delivery. Overall, this study found that both professionals had positive perceptions of each other compared to the study by Naidoo and Bühler (2009), which found that more chiropractic than physiotherapy students had positive perceptions of the other profession. Perhaps this may be because of improved perceptions of physiotherapists and chiropractors over the past years.

Based on the knowledge, attitudes and perceptions of this study, there is evidence that there is minimal ‘animosity’ between these two professionals. There seems to be an inter-professional acceptance, respect and a possibility of collaboration between physiotherapists and chiropractors. This will prevail where relationships between healthcare professionals display inter-professional collaboration, acceptance of their skills and respect for each other during the co-management of patients (Slabbert 2014). The participants in the study support this, as some physiotherapists and chiropractors are already working in close collaboration with each other. This augurs well for patients and healthcare delivery as it will reduce ‘professional individualism’ and facilitate inter-professional collaboration through education and exposure (Ellapen et al. 2018; Reeves et al. 2013). Additionally, a physiotherapist may be more likely to develop a positive inter-professional relationship with a chiropractor if they practise in the private sector or adopt the biopsychosocial model of health, which embraces a holistic approach to patient management. Therefore, although there is a referral relationship between some chiropractors and physiotherapists, this could be augmented by educational institutions which promote knowledge, positive attitudes and perceptions, without which inter-professional referrals to each other would be limited.

Some physiotherapists, mainly in the public sector, indicated poor inter-professional relationships and limited knowledge about chiropractors as they did not engage and had limited interactions and collaboration. The authors are aware that this is a challenge as there are no chiropractors in the public health sector. Perhaps inter-professional interactions and education at the tertiary level could improve this aspect because physiotherapists displayed positive attitudes and perceptions towards chiropractors, reinforcing the association between knowledge, attitudes and perceptions. Responses to an open-ended question related to the non-referral of chiropractors to physiotherapists cited no contact with the other professional and limited patient finances to pay for alternate treatments from professionals. Physiotherapists indicated that poor inter-professional relationships with chiropractors, limited scientific evidence and knowledge of chiropractic, and the absence of chiropractic from the public health sector were some of the reasons that they did not refer patients to chiropractors.

The authors concede that this study has limitations. Firstly, in general, electronic questionnaires have a low response rate as questionnaires sometimes end up as spam mail or participants do not complete these questionnaires. Secondly, our study was conducted at the height of the COVID-19 pandemic. It may have impacted the participants’ ability to access the questionnaires because during the ‘lockdown’, many practitioners (except those in emergency care) may not have been in practice. Although the questionnaires were distributed nationally, the chiropractors were mainly from Gauteng and KwaZulu-Natal. The authors assume this could be attributed to these provinces having two institutions that offer chiropractic programmes. Therefore, the responses from chiropractors could not be generalised to all chiropractors in South Africa.

Based on the above limitations, future research is recommended to determine the value of chiropractors for the public health sector. It would also benefit educational institutions offering physiotherapy and chiropractic programmes to explore strategies to strengthen inter-professional relationships and collaboration between these professionals. The authors believe that the strength of this largely quantitative study is that it was a preliminary and latest study to determine the knowledge, attitudes and perceptions between physiotherapists and chiropractors. Therefore, it offers a starting point for other studies between physiotherapists and chiropractors related to each other’s knowledge, attitudes and perceptions in South Africa. This could strengthen future inter-professional relationships and collaboration between professionals and augment healthcare to patients.

## Conclusion

This is the first study in South Africa on this topic, and the authors tried to gather information from a broad sample of physiotherapists and chiropractors. However, although the response rate was limited, the authors believe that the sample is representative of physiotherapists and chiropractors in South Africa. This is based on the high response rate of physiotherapists and chiropractors in private practice, where predominantly competition for patients exists as there are currently no posts for chiropractors in public health services. Therefore, our study’s limited but representative sample of data indicated that physiotherapists and chiropractors demonstrated good knowledge, mainly positive attitudes and perceptions of each other’s professional practice in South Africa. It is evident that the professionals acknowledge the value of each other, but this could be encouraged and enhanced by professional bodies and institutions offering these courses. This will strengthen and encourage the development of a multidisciplinary team, enabling each profession to ‘share’ their workloads to provide holistic, patient-centred healthcare. Based on this study, positive responses and relevant data, the authors believe that perhaps the anecdotal perception of ‘animosity’ between chiropractors and physiotherapists may not exist or be valid.
